# Biotic Yield Losses in the Southern Amazon, Brazil: Making Use of Smartphone-Assisted Plant Disease Diagnosis Data

**DOI:** 10.3389/fpls.2021.621168

**Published:** 2021-04-15

**Authors:** Anna C. Hampf, Claas Nendel, Simone Strey, Robert Strey

**Affiliations:** ^1^Leibniz Centre for Agricultural Landscape Research (ZALF), Müncheberg, Germany; ^2^Albrecht Daniel Thaer Institute of Agricultural and Horticultural Sciences, Humboldt-Universität zu Berlin, Berlin, Germany; ^3^Institute of Biochemistry and Biology, University of Potsdam, Potsdam, Germany; ^4^Progressive Environmental and Agricultural Technologies (PEAT) GmbH, Hannover, Germany

**Keywords:** plant pathology, animal pests, pathogens, machine learning, digital image processing, disease diagnosis, crowdsourcing, crop losses

## Abstract

Pathogens and animal pests (P&A) are a major threat to global food security as they directly affect the quantity and quality of food. The Southern Amazon, Brazil’s largest domestic region for soybean, maize and cotton production, is particularly vulnerable to the outbreak of P&A due to its (sub)tropical climate and intensive farming systems. However, little is known about the spatial distribution of P&A and the related yield losses. Machine learning approaches for the automated recognition of plant diseases can help to overcome this research gap. The main objectives of this study are to (1) evaluate the performance of Convolutional Neural Networks (ConvNets) in classifying P&A, (2) map the spatial distribution of P&A in the Southern Amazon, and (3) quantify perceived yield and economic losses for the main soybean and maize P&A. The objectives were addressed by making use of data collected with the smartphone application *Plantix*. The core of the app’s functioning is the automated recognition of plant diseases via ConvNets. Data on expected yield losses were gathered through a short survey included in an “expert” version of the application, which was distributed among agronomists. Between 2016 and 2020, *Plantix* users collected approximately 78,000 georeferenced P&A images in the Southern Amazon. The study results indicate a high performance of the trained ConvNets in classifying 420 different crop-disease combinations. Spatial distribution maps and expert-based yield loss estimates indicate that maize rust, bacterial stalk rot and the fall armyworm are among the most severe maize P&A, whereas soybean is mainly affected by P&A like anthracnose, downy mildew, frogeye leaf spot, stink bugs and brown spot. Perceived soybean and maize yield losses amount to 12 and 16%, respectively, resulting in annual yield losses of approximately 3.75 million tonnes for each crop and economic losses of US$2 billion for both crops together. The high level of accuracy of the trained ConvNets, when paired with widespread use from following a citizen-science approach, results in a data source that will shed new light on yield loss estimates, e.g., for the analysis of yield gaps and the development of measures to minimise them.

## Highlights

- ConvNets were trained to identify 420 crop disease classes under diverse conditions.
- Crowdsourcing can significantly improve the data basis for algorithm training.
- Expected yield losses to pests and diseases in the Southern Amazon are below global estimates.
- Annual soybean and maize yield losses to pests and diseases each amount to 3.75 million tonnes.
- Citizen science data can help to identify yield gaps and advance the field of crop loss research.

## Introduction

Pathogens and animal pests (P&A) are major challenges to global food security, directly affecting the quantity (reduced productivity) and quality (e.g., reduced content of valuable nutrients, poorer market quality, and inferior storage characteristics) of food (Oerke, 2006)^1^. They can cause devastating yield losses, leading to malnutrition and starvation, as several examples in history have shown (e.g., the Irish Potato Famine (1845–49), caused by potato leaf blight; and witches’ broom disease, *Moniliophthora perniciosa*, which destroyed Brazil’s leading position in world cocoa production). Globally, direct yield losses to P&A were estimated to range between 20 and 30% for major food and cash crops (Oerke and Dehne, 2004; Oerke, 2006; Savary et al., 2019). Besides these direct effects on food provision, P&A also have indirect effects on the environment (e.g., pesticide use, soil contamination), public health (e.g., mycotoxin contamination) and the economic performance of rural communities (Savary et al., 2012).

The Southern Amazon (specifically, the states of Mato Grosso and Pará) is Brazil’s largest domestic producing region of cotton (64% of national output), maize (34%) and soybeans (28%) (CONAB, 2019). High annual rainfalls and relatively long wet seasons with reliable onset dates allow for the cultivation of two crops in one season (Arvor et al., 2014). Early maturing soybean cultivars are grown at the onset of the rainy season and are either followed by maize or cotton. The high production intensity as well as the warm and humid climate, however, make the region susceptible to the outbreak and spread of P&A. Soybean, maize and cotton production are expected to decrease by 30–40% if farmers do not make use of pesticides to control major P&A (CEPEA, 2019). One of the largest threats to crop production in the Southern Amazon is the fungus *Phakopsora pachyrhizi*, commonly known as Asian soybean rust, causing yield losses of up to 90% (Godoy et al., 2016). Since its first occurrence in Brazil in the early 2000s, the fungus has caused annual yield losses in the range of 360,000–4.6 million tonnes, and economic losses (grain loss +pest control costs) of approximately US$0.18–2.38 billion per year (Godoy et al., 2016).

However, although P&A can cause immense crop damage and economic losses, there are very few systematic research and monitoring programmes on the impact of P&A on crop performance and their spatial distribution. Yield loss data is often based on a limited number of site-specific tests or a particular pathogen over one season. As a result, there has been a persistent and chronic lack of knowledge on the frequency and extent of crop losses caused by plant diseases (Esker, 2012; Nelson, 2017). Moreover, biotic yield losses are largely ignored in yield gap analysis. Yield gaps are an essential concept in crop loss research, defined as the difference between potential yields and actual yields (van Ittersum et al., 2013). While yield losses due to nutrient- and water deficiency were extensively explored using crop modelling, such studies for P&A or weeds are still missing. One major challenge to quantifying P&A-related yield losses is the extremely large diversity of plant diseases, the diversity of life cycles of these organisms and the enormous number of interactions that may exist between P&A and their host crops (Donatelli et al., 2017; Savary et al., 2018).

Various methodological approaches have been used to identify P&A and to quantify associated yield losses, including field experiments (Savary et al., 2016), expert surveys (Savary et al., 2019), simulation modelling (Bregaglio and Donatelli, 2015; Donatelli et al., 2017), remote sensing (Mahlein, 2016), image recognition techniques (Barbedo, 2013; Barbedo et al., 2016), and deep learning models (Boulent et al., 2019). Deep learning model and in particular convolutional neural networks (ConvNets) have recently achieved impressive identification performances in various visual classification tasks, such as the automatic identification of plants and animals (LeCun et al., 2015; ImageCLEF, 2018). Due to their capacity to generalise, they can overcome many of the challenges (e.g., diseases with similar symptoms, multiple simultaneous disorders in a single plant) faced by traditional classification methods (e.g., thresholding, fuzzy classifier, feature-based rules), which appear to be either too specific (identifying just a small number of pathogens) or too sensitive (functioning only under strict operation conditions) (Barbedo, 2013; Boulent et al., 2019).

Several studies demonstrated that ConvNets can be trained to identify a large number of different plant-disease combinations with an accuracy of 85–99% (Mohanty et al., 2016; Ferentinos, 2018; Boulent et al., 2019). The accuracy of these models, however, drastically fell to 25–30% when they were tested on images taken under conditions other than the training dataset (Mohanty et al., 2016; Ferentinos, 2018). The acquisition of a large, verified database with P&A images from different geographic locations as well as the maximisation of real-condition images in the training dataset are two of the major challenges to further improving ConvNets’ performance (Mohanty et al., 2016; Barbedo, 2018b; Ferentinos, 2018). Integrated into mobile devices such as smartphones, ConvNets can be turned into valuable decision support tools for farmers, allowing for plant disease diagnosis on a massive—indeed global—scale (Hughes and Salathe, 2015; Mohanty et al., 2016; Ferentinos, 2018). For instance, Picon et al. (2019) implemented their trained model into various mobile devices and obtained balanced accuracies of 86 and 98% for two different wheat diseases. In another study, Ramcharan et al. (2019) deployed a ConvNet in a mobile app to identify three different cassava diseases in Tanzania but reported a 32% drop in the classification performance when shifting from the test dataset to real-world images.

To sum up, the most important research gaps are a lack of data on the spatial distribution of plant diseases and associated yield losses in the Southern Amazon, a lack of a large verified database for the training and further improvements of ConvNets and a lack of implementation of deep learning technologies for the automated recognition of plant diseases into a practical tool for farmers and/or extension workers. The study seeks to address these research gaps by targeting the following objectives:

(i) Evaluating the performance of ConvNets in classifying P&A
(ii) Mapping the spatial distribution of P&A in the Southern Amazon, Brazil
(iii) Quantifying perceived yield losses for main soybean and maize diseases
(iv) Discussing the potential benefits and limitations of an automated plant disease classification and possible implications for the field of crop loss research.

The objectives were addressed in a joint effort by the Leibniz Centre for Agricultural Landscape Research (ZALF) and PEAT GmbH (Progressive Environmental and Agricultural Technologies). In 2016, PEAT launched *Plantix*, a mobile decision support application for farmers, extension workers and gardeners that uses image recognition and deep learning to diagnose P&A. As part of this study, the *Plantix* library was expanded to include P&A common to the (sub)tropical environment of the Southern Amazon and a 3 months field test was carried out in 2016 to capture field-condition images and to test and promote the app *in situ*. Since then, *Plantix* users have captured more than a million images of P&A in Brazil.

## Materials and Methods

### Study Area

**FIGURE 1:**
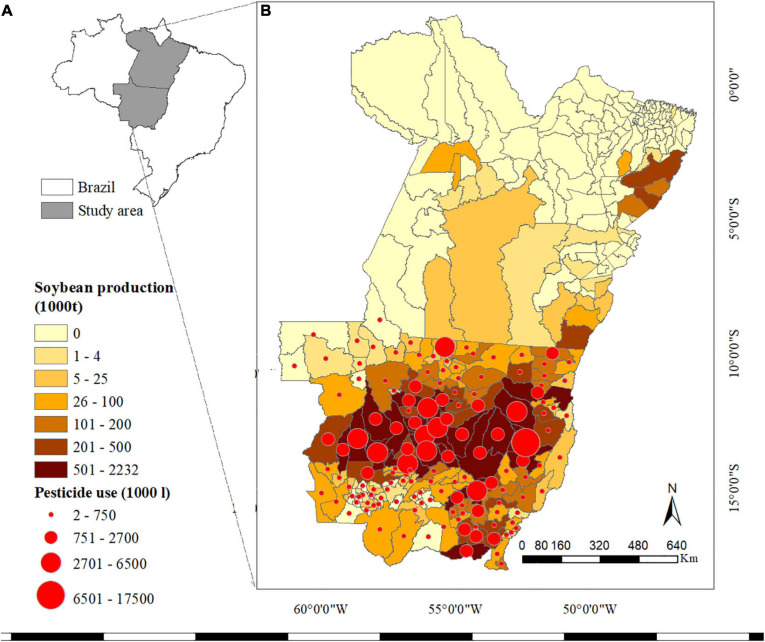
**(A)** Study of area within Brazil. **(B)** Soybean production by municipality in Mato Grosso and Pará in thousands of tonnes in 2018, as well as pesticide use in litres in MT. IBGE (2019b), INDEA (2020). Maps created using ArcMap 10.6.1.

The states of Mato Grosso (MT) and Pará (PA)—located in the Southern Amazon of Brazil (Figure 1A)—are dominated by highly industrialised agricultural systems, mainly consisting of soybean-maize and soybean-cotton rotations. In 2019, farmers in MT produced approximately 32, 31, and 4.5 million tonnes of soybean, maize and cotton (seeds and lint) on 9.6, 4.9, and 1 million ha (Mha) of cropland, respectively (Figure 1B; CONAB, 2020). The climate in the study area is sub(tropical), with pronounced dry and wet seasons and annual precipitation rates ranging from approximately1,000 mm in South MT to over 3,000 mm in Northern PA (INMET, 2019). However, the warm and humid climate, as well as changes in the production system (e.g., expansion of the agricultural frontier northward, extended sowing periods, lack of rotation) have led to a high incidence and spread of P&A in the study area (Godoy et al., 2016; Fundação, 2019). The main diseases affecting agricultural production in the study area are Asian soybean rust (in soybean); common and tropical rust (in maize); and anthracnose and *Ramularia* blight (in cotton) (ABRAPA, 2011; Fundação, 2019). The most damaging pests are those that feed on multiple crops (polyphagous pests) and disperse across fields and over extended periods, such as the lesser cornstalk borer (*Elasmopalpuslignosellus*), the cotton bollworm (*Helicoverpa zea*, also known ascorn earworm), and the fall armyworm (*Spodoptera frugiperda*) (Fundação, 2019). A detailed overview of the main P&A affecting agricultural production in the study area is given in Supplementary Tables 1,2. To reduce the impact of P&A on crop production, genetically modified (GM) crops have been increasingly grown in Brazil. In the 2017–18 cropping season, insect-resistant seeds, herbicide-tolerant seeds or a combination of both were planted on 97, 91, and 84% of soybean, maize (second season) and cotton fields, respectively (Céleres, 2018). Likewise, the use of pesticides in Brazil increased from approximately50 million tonnes in 1990 to 378 million tonnes in 2017 (FAOSTAT, 2019), with MT reporting the largest amount of pesticide use (Pignati et al., 2017). The costs of pesticides (fungicides, herbicides and insecticides) have been estimated to account for 16, 9, and 27% of the total production costs for soybean, maize and cotton, respectively (CEPEA, 2019).

### The *Plantix* Application and Its Workflow

**FIGURE 2:**
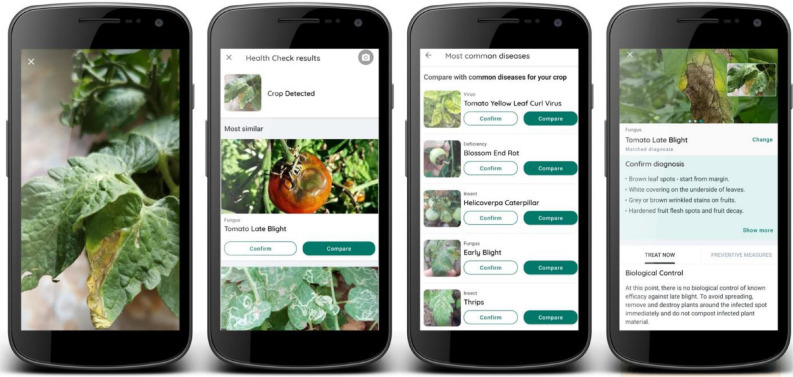
Function flow of the smartphone application Plantix: (1) The user takes a picture of the diseased plant organ. (2) ConvNets classify the image. (3) The user confirms or rejects the diagnosis. (4) The app displays additional information on symptoms, preventive measures and P&A control mechanisms.

Georeferenced images of P&A were collected by users of the *Plantix* smartphone application. The application was released by PEAT in 2016 and is freely available in different languages for any smartphone using the Android operating system. The core function of the app is the automated classification of P&A using ConvNets and involves four steps: (1) taking a picture of the infected plant; (2) classifying the image using several ConvNets; (3) confirming or rejecting the diagnosis by the user; and (4) receiving further information on causes, preventive measures and control options (Figure 2). When taking a picture of a diseased plant in the field, the user can upload the image either directly to a remote server, or the image can be stored on the smartphone and uploaded as soon as a functioning internet connection is available. This enhances the app’s usability in rural and remote areas with low mobile internet connectivity. Once uploaded, the image is classified using multiple ConvNets (one network to determine if the image contains a relevant crop or no plant at all (e.g., an object); one network to classify the crop type; and one to classify the disease). Then, the most similar crop disease combinations (further referred to as “classes”) are displayed to the user and ranked according to their softmax probabilities (see section “Convolutional Neural Networks and Softmax Probability”). Based on this probability ranking as well as a symptom description and reference images for comparison, the user can either confirm or reject the diagnosis. Once the diagnosis is confirmed, the user receives further information on causes, preventive measures, and biological or chemical treatment options. The app can also be used as an offline library, which currently (as of June 2020) contains a description of 592 P&D (267 fungal diseases, 191 insects, 51 bacteria, 51 viruses, 21 mites, and 11 deficiencies). Currently, the ConvNets can automatically detect 231 plant diseases and deficiencies on 49 different species, resulting in a total of 420 classes. Although deficiencies can also be detected, the focus of the application and this study is on P&A.

### Convolutional Neural Networks and Softmax Probability

Image classification in *Plantix* is done via ConvNets, a type of deep neural network, which processes data that comes in the form of an array—for example, a colour image composed of three 2D arrays containing pixel intensities in the three colour channels (LeCun et al., 2015). The architecture of a typical ConvNet mainly consists of convolutional layers and pooling layers. The role of the convolutional layers is to detect local conjunctions of features from the previous layer, whereas the role of the pooling layers is to merge semantically similar features into a single one (LeCun et al., 2015). Multiple ConvNets, such as GoogleNet, AlexNet, and ResNet, have been trained and tested for the classification of plant diseases (Boulent et al., 2019). Other networks, such as EfficientNet (source code/weights) were especially designed for the use on mobile devices. EfficientNet achieves state-of-the-art accuracy with fewer parameters and fewer number of floating-point operations (FLOPs) than other current ConvNets (Tan and Le, 2019; Tan et al., 2019). Due to their high accuracy, this study trained and tested ConvNets using the EfficientNet architecture on a large crowdsourced image database held by PEAT.

To facilitate the interpretation of the network’s output, the convolutional and pooling layers are followed by a fully connected layer. The logits contained in this last layer are converted into probabilities using an activation function, most commonly softmax. Softmax normalises the input array into a scale between 0 and 1, with the sum of the softmax output resulting in 1 (Sharma et al., 2020). In multi-class classification, which is the case of plant disease recognition in *Plantix*, the output of the softmax activation function is given as a vector with probabilities for each class, e.g., [0.2, 0.6, 0.1, …]. The class with the highest probability among all the distributed probabilities is the top-1 prediction. *Plantix* displays the top-1 prediction to the user as the most likely disease, but other predicted classes with lower probabilities (top-2, top-3) can be shown on lower-ranking positions, thus serving as a decision support tool. Although the prediction probability from a softmax distribution has a poor direct correspondence to confidence, correctly classified examples tend to have a greater maximum softmax probability than erroneously classified or out-of-distribution examples (Hendrycks and Gimpel, 2017). Therefore, in this study, only images with a top-1 softmax probability above 0.5 for both the predicted crop and disease were retained in the final dataset.

### Model Training and Testing

The ConvNets implemented in *Plantix* were trained and tested on a large crowdsourced image database, collected either directly by *Plantix* users or by agronomists. While agronomists helped to gather images of less frequent diseases, a high share of images directly collected by *Plantix* users increases the diversity in the image dataset in terms of e.g., image quality, geographic location and smartphone devices. The larger the diversity in the image dataset and the better it reflects the reality of the operational environment, the greater the robustness of the trained model (Barbedo, 2018b; Boulent et al., 2019). Images used for model training and testing were not only collected in Brazil (see section “Fieldwork in Mato Grosso”) but also in other world regions (e.g., Germany, India). All images were either directly annotated by agronomists or annotated and validated afterward by plant experts. The final image dataset was split into a training (2/3) and testing (1/3) subset. Using transfer learning, one ConvNet was trained to classify species (crop ConvNet) and another one to classify P&D and deficiencies (disease ConvNet). The ConvNets are trained with a cosine annealing learning rate over 20 epochs. The total time for training on a machine using two Nvidia GeForce RTX 2080 Ti GPUs is 28 h.

The performance of the ConvNets in identifying plant diseases was assessed by comparing the predicted label (ConvNet classification) to the actual label (expert classification) for each element of the test dataset and calculating the three following evaluation metrics: (1) precision, (2) recall, and (3) F_1_ score. Also, the proportion of images where the correct class was among the top-3 predicted classes was calculated. “Precision” designates the number of images correctly labelled as belonging to the positive class (true positives) divided by the total number of images labelled as belonging to the positive class (sum of true positives and false positives). “Recall” is defined as the number of true positives divided by the total number of images that actually belong to the positive class (sum of true positives and false negatives). The F_1_ score is the harmonic mean of the “precision” and the “recall” figures (Powers, 2011). All metrics are based on the binary confusion matrix (Table 1). Table 2 provides an overview of how to calculate each metric.

**TABLE 1:** Confusion matrix for a classification task.

		**Prediction (ConvNet classification)**	
		**Positive**	**Negative**
Actual (expert classification)	Positive	True positive (Tp)	False positive (Fp)
	Negative	False negative (Fn)	True negative (Tn)
			

**TABLE 2:** Formula of evaluation metrics used to assess ConvNet performance.

***N*°**	***Evaluation indices***	***Formula***
*1*	Precision	$\frac{T\,p}{T\,p+T\,p}$
*2*	Recall (sensitivity)	$\frac{T\,p}{T\,p+F\,n}$
*3*	F_1_ score	$2*\frac{p\,r\,e\,c\,i\,s\,i\,o\,n*r\,e\,c\,a\,l\,l}{p\,r\,e\,c\,i\,s\,i\,o\,n+r\,e\,c\,a\,l\,l}$

### Fieldwork in Mato Grosso

Three months of fieldwork (from September to December 2016) was carried out in MT to collect training images and to promote the app among farmers and their organisations. Before the fieldwork, an online survey was conducted among local agronomists and plant experts to identify the main soybean, maize and cotton plant diseases common to the Southern Amazon. The online survey asked agronomists to rank a literature-based pre-selection of P&A according to their importance and/or to name additional P&A. The survey was sent to agronomists of the Brazilian Agricultural Research Corporation (Embrapa), as well as to universities and research institutes specialising in agronomy. The P&A they identified were incorporated into the *Plantix* library with a description of their symptoms as well as preventive measures and control options. Besides, the library and menu of *Plantix* were translated into Portuguese.

During fieldwork, more than 50 farms in Southeast and Central Mato Grosso were visited to test *Plantix* in the field and collect images that could be used for model training. One crucial element of the fieldwork was to advertise the app among farmers, research institutes, students and the general public to ensure a large engagement in the crowdsourcing project. Advertising materials were distributed at universities and research institutes and sent to public and private farmers’ organisations, such as Aprosoja and the Mato Grosso Research, Assistance and Rural Extension Company (EMPAER), as well as to more than 200 local offices of the Rural Workers’ Union (FETAGRI) and the Rural Union (FAMATO). One example of these advertising materials is shown in Supplementary Figure 1.

### Survey on Perceived Yield Losses and Yield Loss Estimates at the State Level

Besides the above mentioned online survey, a second survey was conducted during the cropping season 2016–17 to gather information on perceived yield losses to P&A. This survey was directly included in an “expert” version of *Plantix*, which was distributed exclusively among agronomists and other plant experts. When taking a picture of a diseased plant, the agronomists were asked to roughly estimate expected (future) yield losses. Six different answer ranges were possible: 0–5, 5–10, 10–20, 20–50, 50–70, and more than 70%. The reason to limit its distribution to plant experts was to ensure the highest possible data quality. However, this also limited the spatial coverage of the survey and most yield losses estimates were provided for Central MT, causing a potential location bias.

To get an approximation of possible yield and economic losses at the municipality and state level, the study assumed that yield loss estimates provided for Central MT would be representative of other production sites in the Southern Amazon. Hence, the expert-based average yield loss estimates of each disease were merged with the kernel density map of the respective disease (see sections “Description of Cleaned-Up Dataset”, “Spatial Distribution” and “Expected Yield Losses”), resulting in spatial yield loss maps of the most important soybean and maize P&A. The mean of these spatial yield loss maps was taken for both soybean and maize and the expected percentage yield losses per municipality were estimated. Next, data on absolute crop production between 2016 and 2018 at the municipality level (IBGE, 2019b) and expected percentage yield losses per municipality were used to calculate absolute yield losses at the municipality and state level. Finally, economic yield losses were estimated assuming average prices of US $355 and $159 per metric tonne for soybean and maize, respectively, for the 2016–2018 cropping seasons in accordance with data from the International Monetary Fund (IMF, 2020).

### Kernel Density Estimation

To visualize the spatial distribution of the predicted P&A, kernel density maps were generated using the *tmaptools* (Tennekes, 2019) package of the open-source software program R (R Core Team, 2020). Kernel density estimation produces a risk map that is interpolated from incident locations in a defined study area. It generalizes or “smooths” discrete data points in a way that a continuous surface area is produced (Hart and Zandbergen, 2014). Here, a 2D kernel density estimator was applied with a bandwidth set to 1/50th of the shorter side of the study area and the resolution of the output raster was set to 1 km^2^. Kernel densities below 0.0001 were set to NA. The output raster were plotted using the R packages *raster* (Hijmans, 2019)*, rasterVis* (Lamigueiro and Hijmans, 2019), and *RColorBrewer* (Neuwirth, 2014).

## Results

### Evaluation of the ConvNets’ Performance

Table 3 summarises the evaluation metrics for the crop and disease ConvNet trained on the *Plantix* image dataset. The evaluation metrics indicate a high performance of the crop and disease ConvNets in identifying 420 classes with a precision of 91.11%, recall of 90.61%, an F_1_ score of 90.86%, and top-3 accuracy of 98.81% (weighted summary; see Table 3). Table 3 also gives the weighted mean of the metrics for 18 maize diseases and 19 soybean diseases, indicating a lower precision for soybean disease detection than for maize.

**TABLE 3:** Summary of evaluation metrics for the crop and disease ConvNet trained using the *Plantix* image dataset.

**Crop**	**Precision**	**Recall**	**F_1_ score**	**Top3**	**N° diseases**
Weighted mean	90.86	91.11	90.61	98.81	420
Mean	88.08	89.63	86.58	97.98	420
Maize	88.40	91.51	89.82	98.99	18
Soybean	78.72	76.64	76.86	96.82	19

**For maize and soybean diseases, weighted metrics are shown.**

### *Plantix* Dataset and Data Cleaning

Between November 2016 and May 2020, *Plantix* users captured about 1.05 million images in Brazil, of which approximately 980,000 showed a plant, whereas the remaining images contain objects. All images containing objects were discarded from the dataset. Most of the images were taken in South Brazil, in the states of Sao Paulo (∼190,000), Santa Catarina (∼172,000) and Minas Gerais (∼2,000). *Plantix* users in the Southern Amazon captured 77,611 P&A images, of which 80% came from MT. From this dataset, all images showing ornamental plants were removed, further reducing the dataset to 70,266 images. Since users often took multiple images at the same location and at the same time, only one image per disease per camera session was allowed. This reduced the dataset to 44,926 images. Finally, the images were filtered according to their softmax probability (see section “Convolutional Neural Networks and Softmax Probability”). All images with a top-1 softmax probability below 0.5 for either the predicted crop or disease type were removed from the dataset, reducing it to 15,921 images. This corresponds to about 20% of the original dataset. The results presented in this study are based on this cleaned-up dataset.

### Description of Cleaned-Up Dataset

**FIGURE 3:**
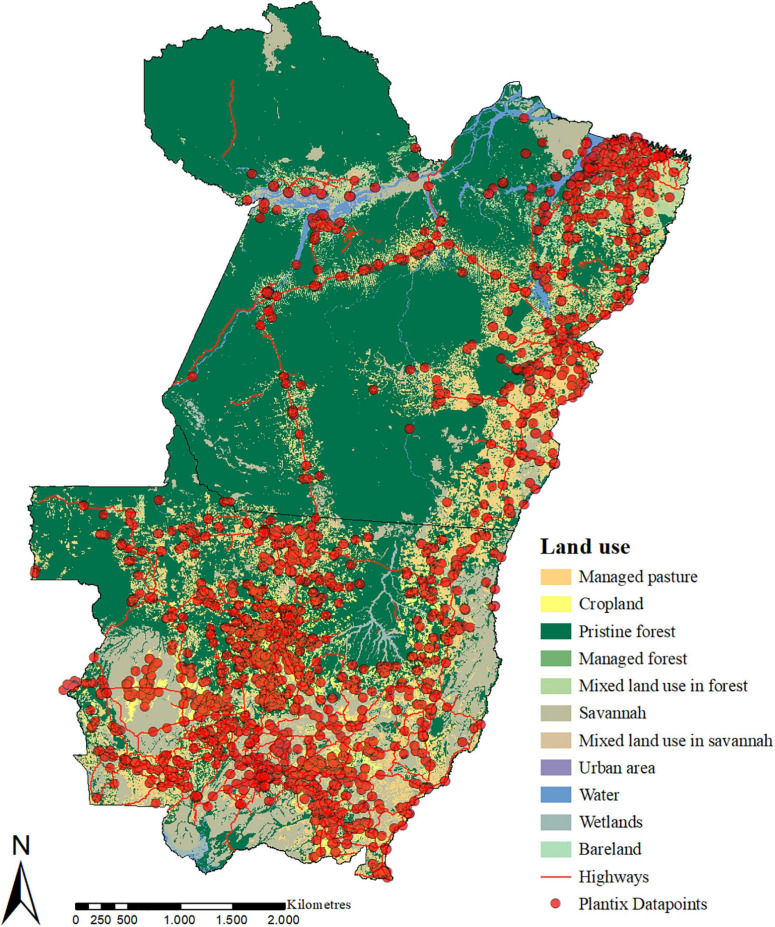
Spatial distribution of P&A images collected by *Plantix* users in Mato Grosso and Pará, Brazil, between November 2016 and May 2020, and the main land-use types in the study area. Source of land use data: IBGE (2018). Map produced using ArcMap 10.6.1.

The cleaned-up dataset contains 15,921 images of P&A and deficiencies that were taken by *Plantix* users in the Southern Amazon between 2016 and 2020. According to the predictions provided by the ConvNets, the dataset holds images of 395 different classes; some, however, are only represented by a few images. A complete list of all classes for which more than 50 pictures were taken can be found in the Supplementary Table 3. The bulk of the images were collected in the main production areas of MT, specifically the central north (Sinop, Sorriso, Lucas do Rio Verde), the southwest (Campo Novo do Parecis, Tangará da Serrá) and the southeast (Primavera do Leste and Campo Verde). Multiple images were also collected along Highway BR-163, which connects Cuiabá (MT) with Santarém (PA) and serves as a soybean export corridor, as well as along the Trans-Amazonian Highway BR-230. Figure 3 shows the spatial distribution of images collected by *Plantix* users in MT and PA between November 2016 and May 2020, as well as the main land-use types in the study area.

### Spatial Distribution

#### Spatial Distribution of Predicted Crop Types

**FIGURE 4:**
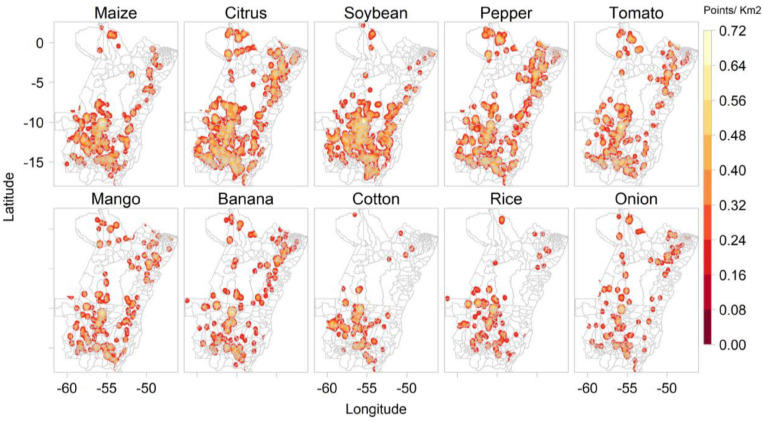
Kernel density of images collected by *Plantix* users in Mato Grosso and Pará, Brazil, between November 2016 and May 2020, according to predicted crop types.

*Plantix* predicted most of the images as showing diseased maize plants (1,973), followed by citrus (1,921), including orange, lemon and tangerine, soybean (1,583), pepper (1,459), tomato (1,390), mango (954), banana (824), cotton (453), rice (442), onion (384), eggplant (369), cucumber (356), papaya (347), and lettuce (313). Figure 4 shows the kernel density of images collected by *Plantix* users in MT and PA between November 2016 and May 2020 according to predicted crop types.

#### Spatial Distribution of Predicted Pest Types

**FIGURE 5:**
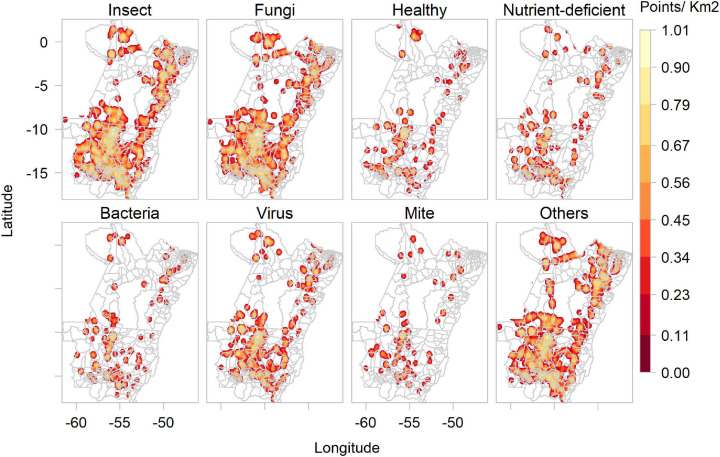
Kernel density of images collected by *Plantix* users and agronomists in Mato Grosso and Pará, Brazil, between November 2016 and May 2020, according to predicted pest types and healthy plants.

The ConvNet that processed images according to disease type predicted most images as showing insects (4,692) or fungal diseases (4,402). Fewer pictures were predicted to show bacteria (899), viruses (577) and mites (366). There were also many images labelled as deficiencies (1,534), e.g., nitrogen, magnesium or iron deficiency. The pathogen class of “others” (219) groups abiotic damage, such as pesticide burn, herbicide damage or sunburn. The “disease” ConvNet also predicted numerous images as containing healthy plants (3,461). Figure 5 shows the kernel density of images collected by *Plantix* users in MT and PA between November 2016 and May 2020, according to predicted pathogen types.

#### Spatial Distribution of Predicted Soybean Pathogens and Animal Pests

**FIGURE 6:**
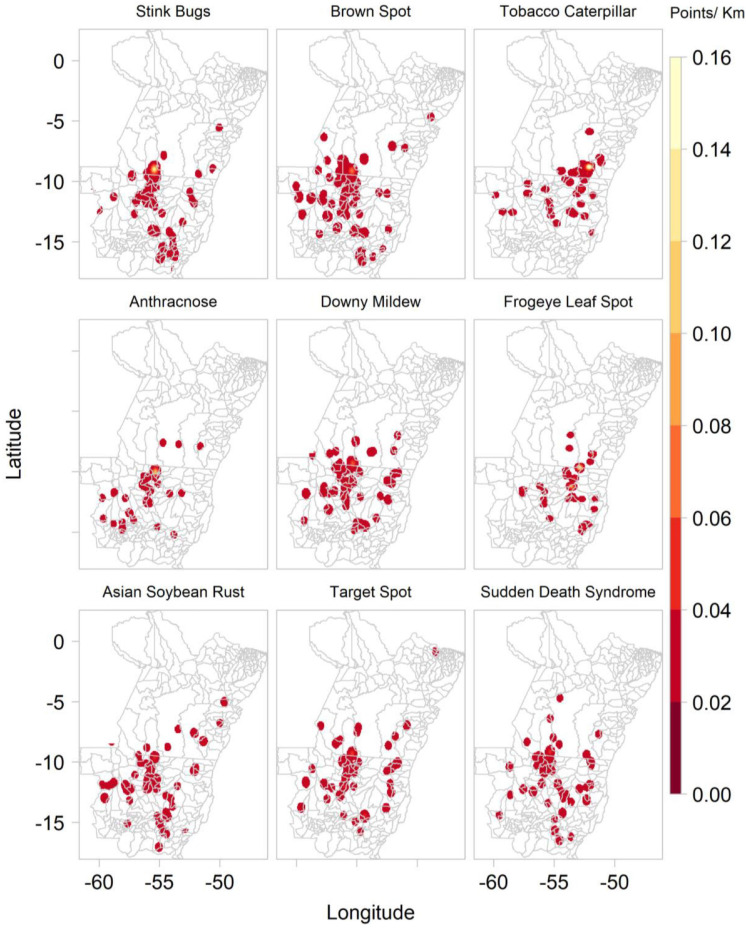
Kernel density of main soybean pathogens and animal pests based on ConvNet predictions provided for images collected by *Plantix* users and agronomists in Mato Grosso and Pará, Brazil, between November 2016 and May 2020.

The “disease” ConvNet interpreted 1,454 out of 1,583 images as showing diseased soybean plants, and the remaining images as healthy soybean plants. These images were classified as stink bugs on soybean (227), brown spot of soybean (159), tobacco caterpillar (138), potassium deficiency (125), anthracnose of soybean (121), downy mildew of soybean (106), frogeye leaf spot (105), Asian soybean rust (94), target spot of soybean (92), and sudden death syndrome (86). Fewer images were predicted to show the fall armyworm (41), the *helicoverpa* caterpillar (35), soybean looper (32), stem rot (25), leaf miner flies (19), boron deficiency (16), spider mites (15), powdery mildew of soybean (9), and castor semi-looper (8). Of the 10 most frequently predicted soybean P&A, all except tobacco caterpillar are mentioned by the Mato Grosso Foundation among the most common soybean diseases found in the Southern Amazon (Supplementary Tables 1, 2). Most images were collected between mid-October and mid-February, which corresponds to the main soybean cropping season, whereas images predicted as containing maize plants were mainly collected either during sowing in March or before harvest in July. The timing of the image data collection is shown in more detail in Supplementary Figure 2. Figure 6 shows the kernel density of the main soybean pests and diseases based on images collected by *Plantix* users in MT and PA between November 2016 and May 2020.

#### Spatial Distribution of Predicted Maize Pathogens and Animal Pests

**FIGURE 7:**
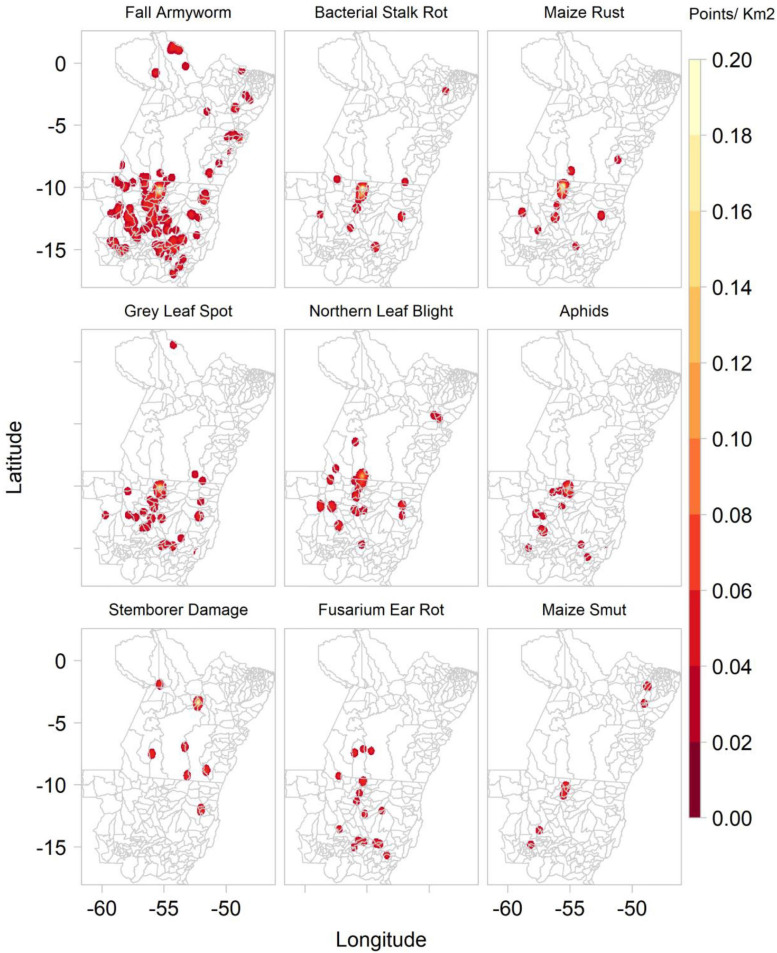
Kernel density of the main maize pathogens and animal pests based on ConvNet predictions provided for images collected by *Plantix* users in Mato Grosso and Pará, Brazil, between November 2016 and May 2020.

Between 2016 and 2020, *Plantix* users captured 1,973 images of maize plants in MT and PA, Brazil, of which 1,854 were interpreted by the “disease” ConvNet to show diseased maize plants and 109 healthy maize plants. According to the system’s predictions, most images were likely to show the fall armyworm (466), bacterial stack rot (233), maize rust (233), grey leaf spot of maize (172), magnesium deficiency (164), northern leaf blight (116), phosphorus deficiency (107), boron deficiency (90), and aphids (75). Fewer images were interpreted to show potassium deficiency (64), nitrogen deficiency (45), stemborer damage (36), fusarium ear rot (23), maize smut (16), and goss wilt (14). Of the 10 most frequently predicted maize P&A, all except northern leaf blight, aphids, maize smut and goss wilt are mentioned by the Mato Grosso Foundation among the most common maize diseases found in the Southern Amazon (Supplementary Tables 1,2). Figure 7 shows the kernel density of the main P&A affecting maize production in the Southern Amazon.

### Expected Yield Losses

#### Expected Yield Losses According to Pathogens and Animal Pests

**FIGURE 8:**
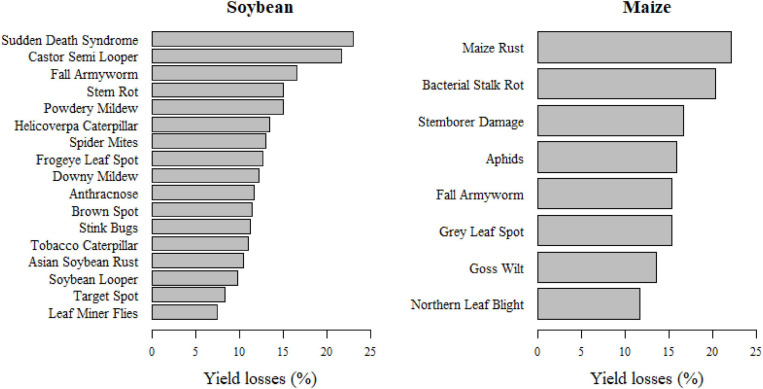
Expected soybean and maize yield losses (%) in Mato Grosso and Pará, Brazil, according to individual pathogens and animal pests.

Agronomists reported expected yield losses for 2,419 images. For soybean and maize, respectively, yield losses were reported for 19 and 13 different classes, including two and five deficiencies, based on 409 and 250 corresponding images. The survey reveals that expected soybean and maize yield losses due to P&A were on average 12.16 and 16%, respectively. However, there were large differences in expected yield losses according to different P&A. For soybean, expected yield losses were highest for the sudden death syndrome (23%), followed by castor semi-looper (21.67%), and fall armyworm (16.6%). Expected maize yield losses were highest for maize rust (22.11%), bacterial stalk rot (20.27%), and stemborer damage (16.6%). Differences in expected maize yield losses according to pest types were rather low, with 18.8% for bacteria, 15.79% for fungi and 15.43% for insects. Likewise, expected soybean yield losses varied little among different pest types, with 13.05% for mites, 12.63% for fungi and 11.67% for insects. Figure 8 shows the expected soybean and maize yield losses according to different P&A.

The study results indicate that the biggest threat to maize production are maize rust, bacterial stalk rot and the fall armyworm, as these three P&A cause high yield losses and are also among the most widespread diseases according to the ConvNet predictions and information provided by the Mato Grosso Foundation (Fundação, 2019). For soybean, the picture is less clear: the Sudden Death Syndrome was reported to cause the highest yield losses, but it was relatively seldom predicted by the ConvNets. P&A like anthracnose, downy mildew, frogeye leaf spot, stink bugs and brown spot seem to pose a greater threat as they are widespread and cause average yield losses of 10–15%. The Asian soybean rust, which was a long time the most severe disease in the study area, seems to be relatively well controlled with average yield losses of 10.5%.

**FIGURE 9:**
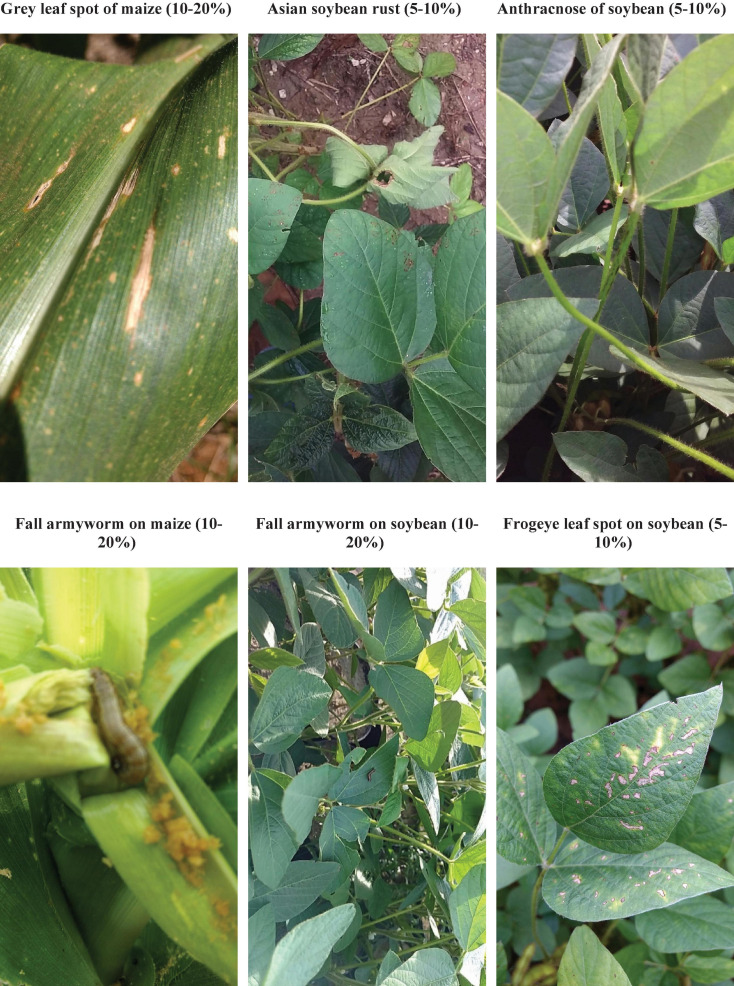
Examples of images of diseased soybean and maize plants and associated expected yield losses provided by agronomists in the state of MT, Brazil, in the 2016–2017 cropping season.

Figure 9 shows examples of P&A images for which agronomists in Mato Grosso provided an estimate of expected yield losses. Most of the images showed diseased plants with mild symptoms and expected yield losses below 20%. The examples demonstrate that the angle, distance and quality of the recorded images may vary considerably and that the background may also be noisy, containing other plant material or soil. Moreover, mild symptoms may be hard to recognise from an image alone, especially non-foliar diseases (e.g., Anthracnose of soybean). These images also exemplify the difficulty plant experts may be confronted with when annotating P&A images.

#### Expected Yield and Economic Losses per Municipality and at the State Level

**FIGURE 10:**
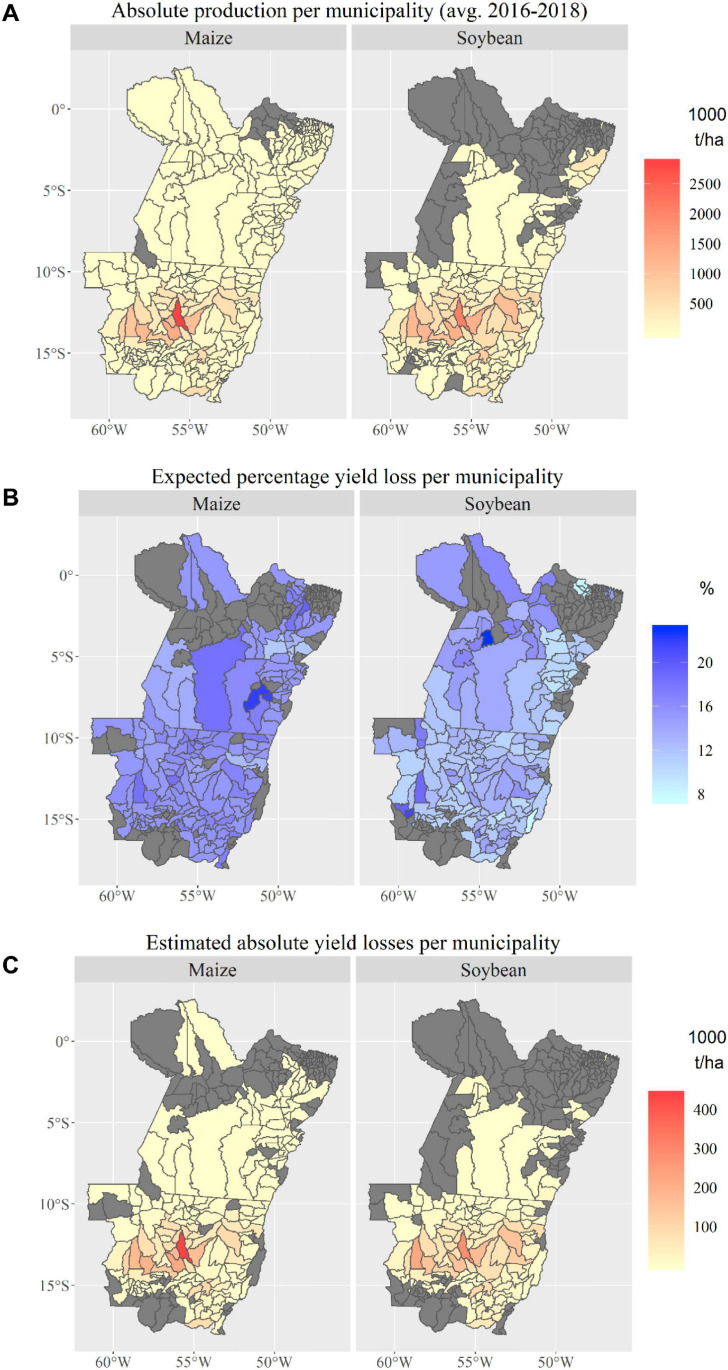
**(A)** Absolute maize and soybean production in municipalities of Mato Grosso and Pará, Brazil; **(B)** expected maize and soybean yield losses (%) due to the most common P&A in the study area; and **(C)** absolute maize and soybean production losses per municipality in Mato Grosso and Pará, Brazil.

Between 2016 and 2018, farmers in MT and PA produced an annual average of approximately 24.6 million tonnes of maize (first and second season) and approximately 31 million tonnes of soybean (IBGE, 2019b). Most of the production originated from Central MT (the municipalities of Sorriso and Nova Mutum, Figure 10A), where also most of the images with estimates on expected yield losses were collected. The expected yield loss estimates at the municipality level reveal that the percentage yield losses range between 7.5 and 23% for soybean and between 11.6 and 22.12% for maize (Figure 10B). Overall, yield loss estimates were available for 78 and 60% of all soybean and maize-producing municipalities, which represent 95 and 97% of the region’s total respective soybean and maize production. The estimation of yield losses at the state level reveals that, on average, 3.74 and 3.75 million tonnes of soybean and maize, respectively, were lost in MT and PA between 2016 and 2018 (Figure 10C). This translates into economic losses of around US $2 billion per cropping season ($1.33 and $0.6 billion for soybean and maize, respectively).

## Discussion

The launch of the *Plantix* mobile application demonstrated that ConvNets trained for the automatic classification of plant diseases are not only ready to be put into operational use, but that such a decision support tool can achieve great attention and widespread use among farmers, generating data that can, in turn, be used by science. Based on the lessons learned from *Plantix* in Brazil, the following section outlines the potential benefits and limitations of an automated plant disease classification via a mobile application, as well as possible benefits for the scientific community—especially in the field of crop loss research.

### Crowdsourcing the Collection of P&A Images

ConvNets—like other supervised machine learning algorithms—require large amounts of human-annotated data to be trained successfully. However, the development of such a human-annotated image database for plant diseases has been one of the major challenges in further improving the performance of ConvNets and making them fully operational in the field (Barbedo, 2018a,b; Boulent et al., 2019). One option to generate such a database is to crowdsource it. PEAT has relied on just such a crowdsourcing process, as the images taken by *Plantix* users constantly add to a database that can—at least in part—be used for model training. The advantage of this crowdsourced image data collection process is that images are acquired at different locations, at different hours of the day, under different meteorological conditions and with different smartphone devices, thus accurately reflecting the reality of the operational environment. The growing field of citizen science could help scientists to crowdsource the collection of P&A images. Citizen science means the involvement of the general public in scientific work, often in collaboration with or under the direction of professional scientists (European Commission, 2013). Citizen science projects can be found in fields ranging from astronomy to medicine and computer science to earth observations, including from the field of plant pathology (Walther and Kampen, 2017; Luigi Nimis et al., 2018; D’Agostino et al., 2020).

### Potential Benefits and Limitations of ConvNet Systems as a Decision Support Tool for Farmers

The implementation of ConvNets trained for the automatic classification of plant diseases into a mobile application proved to be a useful decision support tool for farmers and gardeners. In particular, the high top-3 score (98%) indicates that a diagnosis given by the application can help farmers to identify the correct diseases among a pre-selected list. A simple, easy-to-use and free tool is particularly attractive for small-scale farmers, who often do not have access to agricultural extension services or who lack the financial means for such services. One major advantage of such a tool is that it can be used by anyone (regardless of education level or scouting experience) with an internet-enabled smartphone. Furthermore, the services are immediately available at any time, but leave the user with the final decision of whether and how to protect or treat the plants. The app can also help farmers to reduce the number of pesticide applications by promoting the adoption of non-chemical methods, such as pheromones, biopesticides or the removal and burning of affected plant parts (integrated pest management). Moreover, the functionality of the app can be expanded to include an early warning system by sending push-notifications in case a disease has been spotted in a nearby plot or by recommending fertiliser use and the timing of sowing, among other benefits.

One disadvantage of ConvNets in diagnosing plant diseases is the timing of the detection: Since this form of detection relies on visual symptoms, the earliest possible detection is when symptoms are visible to the human eye and can be recorded by a camera. However, plants may be affected by a pathogen much earlier and consequently react to its presence with, e.g., a reduction in the photosynthesis rate, which induces an increase in fluorescence and heat emission (Martinelli et al., 2014). Therefore, the earliest management of a pest or disease can only happen if there has already been a visual change in the plant material. Moreover, the success and usability of such a tool not only depends on its classification performance but also its availability in different languages and the inclusion of locally relevant diseases. A major hurdle for any system’s use is also the affordability of smartphones and internet connectivity. Although the smartphone penetration rate is increasing rapidly (45% of the Brazilian population actively used a smartphone in 2019; Newzoo, 2019), it is still limited in rural areas and among farm households. According to the latest agricultural census, in MT and PA, only 26% of farmers in MT and only 13% in PA have access to the internet. Even fewer farmers use the mobile internet: 14 and 10% in MT and PA, respectively (IBGE, 2019a). Attempts to distribute such a smartphone app in other countries, e.g., Nigeria, basically failed because too few people own smartphones in rural areas.

### Reducing Yield Losses to Pests and Diseases

The study results indicate that soybean and maize yield losses amount, on average, to 12 and 16%, respectively, and are hence slightly lower than reported yield loss estimates at the global scale of 19 and 21% for these two crops (Oerke, 2006; Savary et al., 2019). This is likely due to the massive and prophylactic application of pesticides in the study area (Pignati et al., 2017; INDEA, 2020). Another explanation for the rather low yield loss estimates is that much of the data was collected in Central MT, where the most productive and experienced farmers are located, and where the cultivation of soybean and maize is an established practice. The study results might, therefore, underestimate yield losses in other municipalities and hence also at the state level. Estimated annual soybean yield losses of 3.74 million tonnes, as well as corresponding economic losses of US $1.33 billion, are within the range of estimates provided by Godoy et al. (2016). The results also indicate that there is a large variation in crop losses due to specific P&A, which is in agreement with the findings of Savary et al. (2019), but a rather low variation in yield losses according to pathogen types (e.g., fungi, bacteria).

P&A are mainly controlled through the intensive use of pesticides in combination with the cultivation of GM crops, which may cause negative effects for human health, such as acute and chronic intoxication (Pignati et al., 2017) and biodiversity loss, as well as the development of pest resistance to pesticides (Karlsson Green et al., 2020). One alternative, holistic approach to combating pests is integrated pest management (IPM), which combines preventive and curative methods, and only applies chemical pesticides when there is an urgent need (Karlsson Green et al., 2020). A field experiment jointly established by the Embrapa and Aprosoja in MT to test the efficacy of IPM demonstrated that areas managed using IPM measures produced the same yield as areas with conventional management, but used approximately 50% less insecticide (Bueno et al., 2020). Despite its large potential to decrease pesticide use as well as production costs, the adoption of IPM in Brazil sharply declined in the 2000s due to the introduction of double-cropping and no-tillage systems (Panizzi, 2013). Reviving the adoption of IPM among farmers through targeted public policies and governmental funding agencies, as well as the adaptation of IPM to new circumstances and production systems, can help to minimise biotic yield losses while maintaining environmental quality.

### Possible Implications for Crop Loss Research and Limitations of This Study

This study contributes to the field of crop loss research by providing probability distribution maps and yield loss estimates for the main soybean and maize P&A of the Southern Amazon, one of Brazil’s—and the world’s—most important agricultural regions. These yield loss estimates fill a major data gap and comprise one of the few spatially explicit available datasets for different P&A in Brazil. The analysis provided here can easily be extended to other crops or world regions as more data becomes available, which in turn will enable future researchers to train the underlying ConvNets for more crop disease combinations. Besides, the georeferenced images can be combined with other spatial data (e.g., climate, soil data) to identify factors influencing the outbreak and spread of diseases (Wieland et al., 2017) and to model and predict their spatio-temporal distribution. The georeferenced images collected by *Plantix* users can also be used for other purposes, such as ground-truth labels for the classification of crops and diseases via satellite images. One example of such an application can be found in Wang et al. (2020), who used the *Plantix* image database and deep learning to map crop types in southeast India. Nonetheless, the probability distribution maps and reported yield loss estimates provided in the present study must be interpreted with caution: despite the data cleaning steps applied, which reduced the original dataset by 80%, the probability distribution maps in this study might be biased, as the collection of data points depended on the number of active users in an area. Although images were collected in almost all crop-producing areas and a great deal of effort was devoted to advertising the application throughout the study area, some diseases might be underrepresented or might not have been captured at all.

## Conclusion

The overall objective of this study was to map the spatial distribution of the main soybean and maize diseases in the Southern Amazon and to quantify the associated yield losses by making use of data collected using the *Plantix* smartphone application. Soybean and maize yield losses to P&A in the Southern Amazon were found to be lower than biotic yield losses reported for these crops in other world regions. A likely explanation is the massive and prophylactic application of large amounts of pesticides in the study area. Integrated pest management can be a sustainable alternative to the intensive use of pesticides, helping to minimise negative outcomes for human health, biodiversity and the environment. ConvNets can aid farmers in the early detection and non-chemical control of P&A, while crowdsourcing may aid researchers in gathering training data that accurately reflects the target operational environment. The high level of accuracy of the trained ConvNets, paired with widespread use through a citizen science approach, provides a unique source of data that allows scientists to get a new angle on yield loss estimates, e.g., for the analysis of yield gaps and the development of measures to minimise them.

## Data Availability Statement

The raw data supporting the conclusions of this article will be made available by the authors, without undue reservation.

## Author Contributions

AH, RS, and CN: conceptualisation. AH and RS: data preparation. AH: methodology and writing—original draft preparation. AH, SS, RS, and CN: writing—review and editing. CN: supervision. All authors have read and agreed on the published version of the manuscript.

## Conflict of Interest

SS and RS are the founders of the company PEAT GmbH. The remaining authors declare that the research was conducted in the absence of any commercial or financial relationships that could be construed as a potential conflict of interest.

## Footnotes

1 The term *crop losses* refers to both quantitative and qualitative losses, whereas the term *yield losses* covers quantitative losses only. The focus of this study is on yield losses.

## References

[B1] ABRAPA (2011). *Algodão no Cerrado do Brasil*, 2nd Edn, ed. Freire E. C. (Goiânia: Mundial Gráfica).

[B2] Arvor D. Dubreuil V. Ronchail J. Simões M. Funatsu B. M. (2014). Spatial patterns of rainfall regimes related to levels of double cropping agriculture systems in Mato Grosso (Brazil). *Int. J. Climatol.* 34 2622–2633. 10.1002/joc.3863

[B3] Barbedo J. G. A. (2013). Digital image processing techniques for detecting, quantifying and classifying plant diseases. *SpringerPlus* 2 660. 10.1186/2193-1801-2-660 PMC3863396 24349961

[B4] Barbedo J. G. A. (2018a). Factors influencing the use of deep learning for plant disease recognition. *Biosyst. Eng.* 172 84–91. 10.1016/j.biosystemseng.2018.05.013

[B5] Barbedo J. G. A. (2018b). Impact of dataset size and variety on the effectiveness of deep learning and transfer learning for plant disease classification. *Comput. Electron. Agric.* 153 46–53. 10.1016/j.compag.2018.08.013

[B6] Barbedo J. G. A. Koenigkan L. V. Santos T. T. (2016). Identifying multiple plant diseases using digital image processing. *Biosyst. Eng.* 147 104–116. 10.1016/j.biosystemseng.2016.03.012

[B7] Boulent J. Foucher S. Theau J. St-Charles P. L. (2019). Convolutional neural networks for the automatic identification of plant diseases. *Front. Plant Sci.* 10:941. 10.3389/fpls.2019.00941 31396250 PMC6664047

[B8] Bregaglio S. Donatelli M. (2015). A set of software components for the simulation of plant airborne diseases. *Environ. Modell. Softw.* 72 426–444. 10.1016/j.envsoft.2015.05.011

[B9] Bueno A. F. Panizzi A. R. Hunt T. E. Dourado P. M. Pitta R. M. Goncalves J. (2020). Challenges for adoption of integrated pest management (IPM): the soybean example. *Neotrop. Entomol.* 50 5–20. 10.1007/s13744-020-00792-9 32737866

[B10] Céleres (2018). *20 Anos da Adoção da Biotecnologia Agrícola no Brasil: Lições Aprendidas e Novos Desafios.* Uberlandia: Celeres.

[B11] CEPEA (2019). *Mensuração Econômica da Incidência de Pragas e Doenças no Brasil: Uma Aplicação Para as Culturas de Soja, Milho e Algodão.* Piracicaba: CEPEA.

[B12] CONAB (2019). *Séries Históricas de Área Plantada, Produtividade e Produção, Relativas às Safras 1976/77 a 2018/19 de Grãos.* Rio Branco: CONAB.

[B13] CONAB (2020). *Séries Históricas de Área Plantada, Produtividade e Produção, Relativas às Safras 1976/77 a 2018/19 de Grãos.* Available online at: https://www.conab.gov.br/info-agro/safras/serie-historica-das-safras?start=20 (accessed Augest 15, 2020).

[B14] D’Agostino D. Law-Green D. Watson M. Novara G. Tiengo A. Sandrelli S. (2020). A citizen science exploration of the X-ray transient sky using the EXTraS science gateway. *Future Gen. Comput. Syst.* 111 806–818. 10.1016/j.future.2019.10.030

[B15] Donatelli M. Magarey R. D. Bregaglio S. Willocquet L. Whish J. P. M. Savary S. (2017). Modelling the impacts of pests and diseases on agricultural systems. *Agric. Syst.* 155 213–224. 10.1016/j.agsy.2017.01.019 28701814 PMC5485649

[B16] Esker P. (2012). Crop loss analysis and global food supply: focusing now on required harvests. *CAB Rev. Perspect. Agric. Vet. Sci. Nutr. Nat. Resour.* 7 1–14. 10.1079/pavsnnr20127052

[B17] European Commission (2013). *Green Paper on Citizen Science. Citizen Science for Europe. Towards a Better Society of Empowered Citizens and Enhanced Research.* Brussels: European Commission.

[B18] FAOSTAT (2019). *Pesticides use Dataset.* Available online at: http://www.fao.org/faostat/en/#data/RP (accessed December 01, 2019).

[B19] Ferentinos K. P. (2018). Deep learning models for plant disease detection and diagnosis. *Comput. Electron. Agric.* 145 311–318. 10.1016/j.compag.2018.01.009

[B20] Fundação M. T. (2019). *Boletim de Pesquisa N°18, 2017/2018.* Rondonópolis: Fundação, MT.

[B21] Godoy C. V. Seixas C. D. S. Soares R. M. Marcelino-Guimarães F. C. Meyer M. C. Costamilan L. M. (2016). Asian soybean rust in Brazil: past, present, and future. *Pesq. Agropec. Bras.* 51 407–421. 10.1590/s0100-204x2016000500002

[B22] Hart T. Zandbergen P. (2014). Kernel density estimation and hotspot mapping. *Policing Int. J. Police Strategies Manag.* 37 305–323. 10.1108/pijpsm-04-2013-0039

[B23] Hendrycks D. Gimpel K. (2017). A baseline for detecting misclassified and out-of-distribution examples in neural networks. *Paper Presented at the 5th International Conference on Learning Representations*, Toulon.

[B24] Hijmans R. J. (2019). *Raster: Geographic Data Analysis and Modeling. R Package Version 3.0-7.* Available online at: https://CRAN.R-project.org/package=raster (accessed June, 2019).

[B25] Hughes D. P. Salathe M. (2015). An open access repository of images on plant health to enable the development of mobile disease diagnostics. *arXiv* [Preprint]. 1511.08060.

[B26] IBGE (2018). *Monitoramento da Cobertura e uso da Terra do Brasil 2014-2016.* Rio de Janeiro: IBGE.

[B27] IBGE (2019a). *Censo Agropecuário: Resultados definitivos 2017.* Available online at: https://censos.ibge.gov.br/agro/2017 (accessed October 25, 2019).

[B28] IBGE (2019b). *Pesquisa Agrícola Municipal. Tabela 5457: Área plantada ou Destinada à colheita, Área Colhida, Quantidade Produzida, Rendimento Médio e Valor da Produção das Lavouras Temporárias e Permanentes.* Available online at: https://sidra.ibge.gov.br/pesquisa/pam/tabelas (accessed November 13, 2019).

[B29] ImageCLEF (2018). *ImageCLEF- Cross-Language Image Retrieval Evaluations: ExperLifeCLEF 2018.* Available online at: https://www.imageclef.org/node/231 (accessed October 10, 2018).

[B30] IMF (2020). *Primary Commodity Price System. Market Prices for Non-Fuel and Fuel Commodities.* Available online at: https://data.imf.org/?sk=471DDDF8-D8A7-499A-81BA-5B332C01F8B9 (accessed August 18, 2020).

[B31] INDEA (2020). *Relatório de Comércio de Agrotóxicos Consolidado.* Available online at: http://www.indea.mt.gov.br/-/6099478-agrotoxicos?ciclo= (accessed April 6, 2020).

[B32] INMET (2019). *Estações e Dados. Banco de Dados Meteorológicos para Ensino e Pesquisa.* Available online at: http://www.inmet.gov.br (accessed May 17, 2019).

[B33] Karlsson Green K. Stenberg J. A. Lankinen A. (2020). Making sense of Integrated Pest Management (IPM) in the light of evolution. *Evol. Appl.* 13 1791–1805. 10.1111/eva.13067 32908586 PMC7463341

[B34] Lamigueiro O. P. Hijmans R. J. (2019). *RasterVis. R Package Version 0.47.*

[B35] LeCun Y. Bengio Y. Hinton G. (2015). Deep learning. *Nature* 521 436–444. 10.1038/nature14539 26017442

[B36] Luigi Nimis P. Pittao E. Altobelli A. De Pascalis F. Laganis J. Martellos S. (2018). Mapping invasive plants with citizen science. A case study from Trieste (NE Italy). *Plant Biosyst.* 153 700–709. 10.1080/11263504.2018.1536085

[B37] Mahlein A. K. (2016). Plant disease detection by imaging sensors – Parallels and specific demands for precision agriculture and plant phenotyping. *Plant Dis.* 100 241–251. 10.1094/PDIS-03-15-0340-FE 30694129

[B38] Martinelli F. Scalenghe R. Davino S. Panno S. Scuderi G. Ruisi P. (2014). Advanced methods of plant disease detection. A review. *Agron. Sustain. Dev.* 35 1–25. 10.1007/s13593-014-0246-1

[B39] Mohanty S. P. Hughes D. P. Salathe M. (2016). Using deep learning for image-based plant disease detection. *Front. Plant Sci.* 7:1419. 10.3389/fpls.2016.01419 27713752 PMC5032846

[B40] Nelson A. (2017). Crop pests: crop-health survey aims to fill data gaps. *Nature* 541:464. 10.1038/541464a 28128234

[B41] Neuwirth E. (2014). *RColorBrewer: ColorBrewer Palettes. R Package Version 1.1-2.*

[B42] Newzoo (2019). *Global Mobile Market Report.* Available online at: https://resources.newzoo.com/hubfs/Reports/2019_Free_Global_Mobile_Market_Report.pdf (accessed August 12, 2020).

[B43] Oerke E. C. (2006). Crop losses to pests. *J. Agric. Sci.* 144:31. 10.1017/s0021859605005708

[B44] Oerke E. C. Dehne H. W. (2004). Safeguarding production—losses in major crops and the role of crop protection. *Crop Protect.* 23 275–285. 10.1016/j.cropro.2003.10.001

[B45] Panizzi A. R. (2013). History and contemporary perspectives of the integrated pest management of soybean in Brazil. *Neotrop. Entomol.* 42 119–127. 10.1007/s13744-013-0111-y 23949744

[B46] Picon A. Alvarez-Gila A. Seitz M. Ortiz-Barredo A. Echazarra J. Johannes A. (2019). Deep convolutional neural networks for mobile capture device-based crop disease classification in the wild. *Comput. Electron. Agric.* 161 280–290. 10.1016/j.compag.2018.04.002

[B47] Pignati W. A. Lima F. Lara S. S. Correa M. L. M. Barbosa J. R. Leao L. (2017). Spatial distribution of pesticide use in Brazil: a strategy for Health Surveillance. *Cien. Saude Colet.* 22 3281–3293. 10.1590/1413-812320172210.17742017 29069184

[B48] Powers D. M. W. (2011). Evaluation: from precision, recall and F-measure to ROC, informedness, merkedness & correlation. *J. Mach. Learn. Technol.* 2 37–63.

[B49] R Core Team (2020). *R: A language and Environment for Statistical Computing.* Vienna: R Foundation for Statistical Computing.

[B50] Ramcharan A. McCloskey P. Baranowski K. Mbilinyi N. Mrisho L. Ndalahwa M. (2019). A Mobile-Based Deep Learning Model for Cassava Disease Diagnosis. *Front Plant Sci* 10:272. 10.3389/fpls.2019.00272 30949185 PMC6436463

[B51] Savary S. Ficke A. Aubertot J.-N. Hollier C. (2012). Crop losses due to diseases and their implications for global food production losses and food security. *Food Secur.* 4 519–537. 10.1007/s12571-012-0200-5

[B52] Savary S. Jouanin C. Félix I. Gourdain E. Piraux F. Willocquet L. (2016). Assessing plant health in a network of experiments on hardy winter wheat varieties in France: multivariate and risk factor analyses. *Eur. J. Plant Pathol.* 146 757–778. 10.1007/s10658-016-0955-1

[B53] Savary S. Nelson A. D. Djurle A. Esker P. D. Sparks A. Amorim L. (2018). Concepts, approaches, and avenues for modelling crop health and crop losses. *Eur. J. Agron.* 100 4–18. 10.1016/j.eja.2018.04.003

[B54] Savary S. Willocquet L. Pethybridge S. J. Esker P. McRoberts N. Nelson A. (2019). The global burden of pathogens and pests on major food crops. *Nat. Ecol. Evol.* 3 430–439. 10.1038/s41559-018-0793-y 30718852

[B55] Sharma S. Sharma S. Althaiya A. (2020). Activation functions in neural networks. *Int. J. Eng. Appl. Sci. Technol.* 4 310–316.

[B56] Tan M. Chen B. Pang R. Vasudevan V. Sandler M. Howard A. (2019). MnasNet: platform-aware neural architecture search for mobile. *Paper Presented at the the IEEE Conference on Computer Vision and Pattern Recognition (CVPR)*, Long Beach, CA.

[B57] Tan M. Le Q. V. (2019). EfficientNet: rethinking model scaling for convolutional neural networks. *Paper Presented at the 36th International Conference on Machine Learning*, Long Beach, CA.

[B58] Tennekes M. (2019). *Tmaptools: Thematic Map Tools. R Package Version 2.0-2.* Available online at: https://github.com/mtennekes/tmaptools (accessed September, 2019).

[B59] van Ittersum M. K. Cassman K. G. Grassini P. Wolf J. Tittonell P. Hochman Z. (2013). Yield gap analysis with local to global relevance—A review. *Field Crops Res.* 143 4–17. 10.1016/j.fcr.2012.09.009

[B60] Walther D. Kampen H. (2017). The citizen science project ‘Mueckenatla’ helps monitor the distribution and spread of invasive mosquito species in Germany. *J. Med. Entomol.* 54 1790–1794. 10.1093/jme/tjx166 29029273 PMC5850493

[B61] Wang S. Di Tommaso S. Faulkner J. Friedel T. Kennepohl A. Strey R. (2020). Mapping crop types in southeast India with smartphone crowdsourcing and deep learning. *Remote Sens.* 12:2957. 10.3390/rs12182957

[B62] Wieland R. Kerkow A. Früh L. Kampen H. Walther D. (2017). Automated feature selection for a machine learning approach toward modeling a mosquito distribution. *Ecol. Modell.* 352 108–112. 10.1016/j.ecolmodel.2017.02.029

