# Supplementary material for: Biotic Yield Losses in the Southern Amazon, Brazil: Making Use of Smartphone-Assisted Plant Disease Diagnosis Data
Source: Front Plant Sci. 2021 Apr 15;12:621168. doi: 10.3389/fpls.2021.621168 (PMC8083370; doi:10.3389/fpls.2021.621168)
Supplement: Supplementary file 1 [file Data_Sheet_1.docx]

**SP-Table 1: Most common pests and their respective scientific names in the Southern Amazon, Brazil. Source: Fundação MT (2019)**

| Crop | Common name | Scientific name |
| --- | --- | --- |
| soybean, maize and cotton | - Burrower bugs - Lesser cornstalk borer - Brown stink bug - Green belly stink bug - Cotton bollworm or corn earworm - Fall armyworm - Soybean looper - Silverleaf whitefly - Broad mite - Red spider mite | - Scaptocoris castanea - Elasmopalpus lignosellus - Euchistus heros - Dichelops melacanthus - Helicoverpa armigera - Spodoptera frugiperda - Chrysodeixis includes - Bemisia tabaci - Polyphagotarsonemus latus - Tetranychus urticae |
| only cotton | - Boll weevil | - Anthonomus grandis |

**SP-Table 2: Most common soybean, maize and cotton diseases, scientific name of pathogens and transmitters and pathogen types in the Southern Amazon, Brazil. Source: Fundação MT (2019)**

| Crop | Common name | Scientific name | Pathogen type |
| --- | --- | --- | --- |
| Soybean | - Asian Soybean Rust - Soybean anthracnose - Soybean Stem Canker - Cercospora leaf blight - Corynespora leaf spot/ Target spot - Frogeye leaf spot - Brown spot - Rhizoctonia Root Rot - Downy mildew - White mold - Southern Blight - Powdery mildew - Charcoal rot - Sudden death syndrome | - Phakopsora pachyrhizi - Colletotrichum truncatum - Phomopsis phaseoli / Diaporthe phaseolorum - Cercospora kikuchii - Corynespora cassiicola - Cercospora sojina - Septoria glycines - Rhizoctonia solani, Thanatephorus cucumeris - Peronospora manshurica - Sclerotinia sclerotiorum - Sclerotium rolfsii - Microsphaera difusa - Macrophomina phaseolina - Fusarium solani f. sp. glycines/ Fusarium tucumaniae | Fungi |
|  | - Soybean mosaic virus | - Transmitted by aphids (Aphidoidea) | Virus |
|  | - Bacterial pustule disease - Wildfire of soybean - Bacterial blight | - Xanthomonas axonopodis pv. glycines - Pseudomonas syringae pv. tabaci - Pseudomonas savastanoi pv. Glycinea | Bacteria |
| Maize | - Maize rust - Tropical rust - Commun rust - Maydis leaf blight - Turcicum leaf blight - Grey leaf spot - Macrospora leaf spot and ear rot - Diplodia ear and stalk rot - Anthracnose leaf blight and stalk rot - Root and stalk rot - Fusarium stalk and ear rot - Macrophomina stalk rot - Giberella stalk rot - Penicillium stalk rot | - Puccinia polysora - Physopella zeae - Puccinia sorghi - Helminthosporium maydis - Exserohilum turcicum - Cercospora zeae-maydis - Stenocarpella macrospora / Diplodia macrospora - Stenocarpella maydis - Colletotrichum graminicola - Phaeocytostroma ambiguum - Fusarium spp. - Macrophomina phaseolina - Gibberella zeae/ Fusarium graminearum - Penicillium spp. ou P. oxalicum | Fungi |
|  | - White spot of maize - Bacterial stalk rot | - P. ananatis - Pseudomonas spp. and Erwina spp | Bacteria |
| Cotton | - Corynespora leaf spot - Myrothecium leaf spot - White mold - Ramularia blight - Fusarium wilt - Anthracnose of cotton - Alternaria leaf spot - Cercospora leaf spot - Leaf blight | - Corynespora casiicola - Myrothecium roridum - Sclerotinia sclerotiorum - Ramularia areola - Fusarium oxysporum f. sp. Vasinfectum - Colletotrichum gossypii - Alternaria spp. - Cercospora spp. - Stemphylium solani | Fungi |
|  | - Bacterial blight of cotton | - Xanthomonas axonopodis | Bacteria |
|  | - Blue disease - Atypical mosaic virus - Common mosaic virus | - Transmitted by whitefly (bemisia tabaci) | Vírus |

| 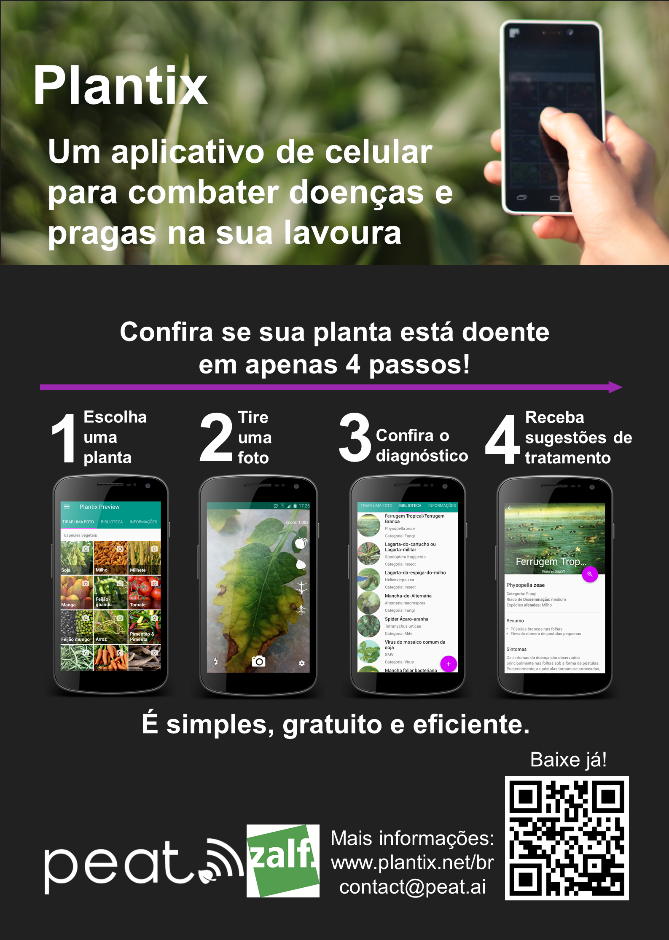 | 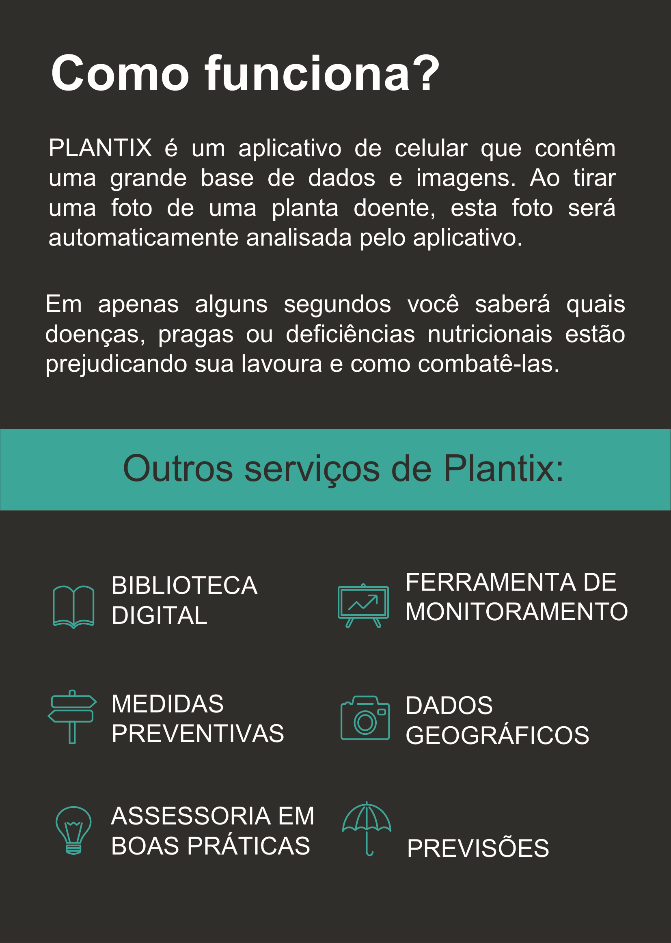 |
| --- | --- |
| SP-Figure 1: Example of advertising material distributed among local research institutes, universities, farmers associations and agencies of the Rural Workers Union and the Rural Union. | |
|  |  |

**SP-Table 3: Most common predictions provided by ConvNets trained on Plantix images database for crops and diseases in Mato Grosso and Pará, Brazil. Only classes for which more than 50 images were taken by *Plantix* users in MT and PA, Brazil, are shown.**

| Crop | Disease, pest or deficiency | Pathogen type | Scientific name | Image freq |
| --- | --- | --- | --- | --- |
| Banana | Panama Disease | Fungi | Fusarium oxysporum | 102 |
| Banana | Yellow and Black Sigatoka | Fungi | Mycosphaerella musicola | 401 |
| Banana | Healthy | Healthy | Healthy | 95 |
| Bean | Healthy | Healthy | Healthy | 54 |
| Bean | Leaf Miner Flies | Insect | Agromyzidae spp. | 62 |
| Cabbage | Black Rot | Bacteria | Xanthomonas campestris pv. campestris | 59 |
| Cabbage | Aphids | Insect | Aphidoidea family | 96 |
| Canola | Black Leg | Fungi | Plenodomus lingam | 51 |
| Citrus | Citrus Canker | Bacteria | Xanthomonas axonopodis pv. citri | 84 |
| Citrus | Zinc Deficiency | Deficiency | Zinc Deficiency | 55 |
| Citrus | Citrus Scab | Fungi | Elsinoe fawcettii | 186 |
| Citrus | Gummosis of Citrus | Fungi | Phytophthora spp. | 89 |
| Citrus | Sooty Mold | Fungi | Capnodium Fumago Scorias spp | 209 |
| Citrus | Healthy | Healthy | Healthy | 199 |
| Citrus | Citrus Blackfly | Insect | Aleurocanthus woglumi | 99 |
| Citrus | Citrus Leaf Miner | Insect | Phyllocnistis citrella | 441 |
| Citrus | Citrus Thrips | Insect | Scirtothrips citri | 73 |
| Citrus | Mealybug | Insect | Pseudococcidae | 159 |
| Coffee | Leaf Miner Flies | Insect | Agromyzidae spp. | 117 |
| Cotton | Alternaria Leaf Spot of Cotton | Fungi | Alternaria macrospora | 91 |
| Cotton | Healthy | Healthy | Healthy | 77 |
| Cotton | Herbicide Burn | Others | Group I herbicides | 92 |
| Cucumber | Downy Mildew of Cucurbits | Fungi | Pseudoperonospora cubensis | 101 |
| Eggplant | Eggplant Lace Bug | Insect | Gargaphia solani | 60 |
| Eggplant | Leaf Miner Flies | Insect | Agromyzidae spp. | 73 |
| Grape | Downy Mildew of Grape | Fungi | Plasmopara viticola | 83 |
| Guava | Mealybug | Insect | Pseudococcidae | 102 |
| Lettuce | Healthy | Healthy | Healthy | 313 |
| Maize | Bacterial Stalk Rot of Maize | Bacteria | Dickeya zeae | 233 |
| Maize | Boron Deficiency | Deficiency | Boron Deficiency | 90 |
| Maize | Magnesium Deficiency | Deficiency | Magnesium Deficiency | 164 |
| Maize | Phosphorus Deficiency | Deficiency | Phosphorus Deficiency | 107 |
| Maize | Potassium Deficiency | Deficiency | Potassium Deficiency | 64 |
| Maize | Common Rust of Maize | Fungi | Puccinia sorghi | 63 |
| Maize | Grey Leaf Spot of Maize | Fungi | Cercospora zeae-maydis | 172 |
| Maize | Northern Leaf Blight | Fungi | Setosphaeria turcica | 116 |
| Maize | Southern Rust of Maize | Fungi | Puccinia polysora | 170 |
| Maize | Healthy | Healthy | Healthy | 119 |
| Maize | Aphids | Insect | Aphidoidea family | 75 |
| Maize | Fall Armyworm | Insect | Spodoptera frugiperda | 466 |
| Mango | Algal Leaf Spot | Additional | Cephaleuros virescens | 51 |
| Mango | Bacterial Black Spot of Mango | Bacteria | Xanthomonas axonopodis pv. mangiferaeindicae | 70 |
| Mango | Sooty Mold | Fungi | Capnodium Fumago Scorias spp | 80 |
| Mango | Mango Malformation | Fungi | Fusarium mangiferae | 54 |
| Mango | Healthy | Healthy | Healthy | 330 |
| Mango | Mango Midge | Insect | Procontarinia | 123 |
| Mango | Thrips | Insect | Thysanoptera | 80 |
| Manioc | Cassava Green Spider Mite | Mite | Mononychellus tanajoa | 188 |
| Melon | Healthy | Healthy | Healthy | 59 |
| Melon | Cucumber Mosaic Virus | Virus | CMV | 144 |
| Onion | Downy Mildew | Fungi | Peronosporales | 65 |
| Onion | Healthy | Healthy | Healthy | 164 |
| Onion | Thrips | Insect | Thysanoptera | 76 |
| Papaya | Powdery Mildew of Papaya | Fungi | Oidium caricae-papayae | 106 |
| Papaya | Papaya Mosaic Virus | Virus | PapMV | 116 |
| Papaya | Ring Spot Virus | Virus | PRSV | 67 |
| Pepper | Bacterial Spot of Pepper | Bacteria | Xanthomonas spp. | 117 |
| Pepper | Magnesium Deficiency | Deficiency | Magnesium Deficiency | 87 |
| Pepper | Nitrogen Deficiency | Deficiency | Nitrogen Deficiency | 72 |
| Pepper | Healthy | Healthy | Healthy | 511 |
| Pepper | Aphids | Insect | Aphidoidea family | 68 |
| Pepper | Chilli Thrips | Insect | Scirtothrips dorsalis | 121 |
| Pepper | Whiteflies | Insect | Aleyrodidae | 145 |
| Pepper | Broad Mite | Mite | Polyphagotarsonemus latus | 56 |
| Pepper | Chilli Leaf Curl Virus | Virus | CLCV | 77 |
| Pomergranate | Healthy | Healthy | Healthy | 63 |
| Rice | Brown Spot of Rice | Fungi | Cochliobolus miyabeanus | 80 |
| Rice | Healthy | Healthy | Healthy | 82 |
| Rose | Healthy | Healthy | Healthy | 177 |
| Soybean | Potassium Deficiency | Deficiency | Potassium Deficiency | 125 |
| Soybean | Anthracnose of Soybean | Fungi | Colletotrichum truncatum | 121 |
| Soybean | Brown Spot of Soybean | Fungi | Septoria glycines | 159 |
| Soybean | Downy Mildew of Soybean | Fungi | Peronospora manshurica | 106 |
| Soybean | Frogeye Leaf Spot | Fungi | Cercospora sojina | 105 |
| Soybean | Soybean Rust | Fungi | Phakopsora pachyrhizi | 94 |
| Soybean | Sudden Death Syndrome | Fungi | Fusarium virguliforme | 86 |
| Soybean | Target Spot of Soybean | Fungi | Corynespora cassiicola | 92 |
| Soybean | Healthy | Healthy | Healthy | 129 |
| Soybean | Stink Bugs on Soybean | Insect | Acrosternum hilare | 227 |
| Soybean | Tobacco Caterpillar | Insect | Spodoptera litura | 138 |
| Strawberry | Nitrogen Deficiency | Deficiency | Nitrogen Deficiency | 63 |
| Strawberry | Healthy | Healthy | Healthy | 87 |
| Tomato | Bacterial Spot of Tomato | Bacteria | Xanthomonas spp. | 252 |
| Tomato | Blossom End Rot | Deficiency | blossom end rot | 55 |
| Tomato | Nitrogen Deficiency | Deficiency | Nitrogen Deficiency | 56 |
| Tomato | Early Blight | Fungi | Alternaria solani | 121 |
| Tomato | Tomato Late Blight | Fungi | Phytophthora infestans | 77 |
| Tomato | Healthy | Healthy | Healthy | 163 |
| Tomato | Leaf Miner Flies | Insect | Agromyzidae spp. | 138 |
| Tomato | Tobacco Caterpillar | Insect | Spodoptera litura | 57 |
| Tomato | Herbicide Growth Damage | Others | Group I herbicides | 53 |
| Tomato | Tomato Yellow Leaf Curl Virus | Virus | TYLCV | 62 |

**
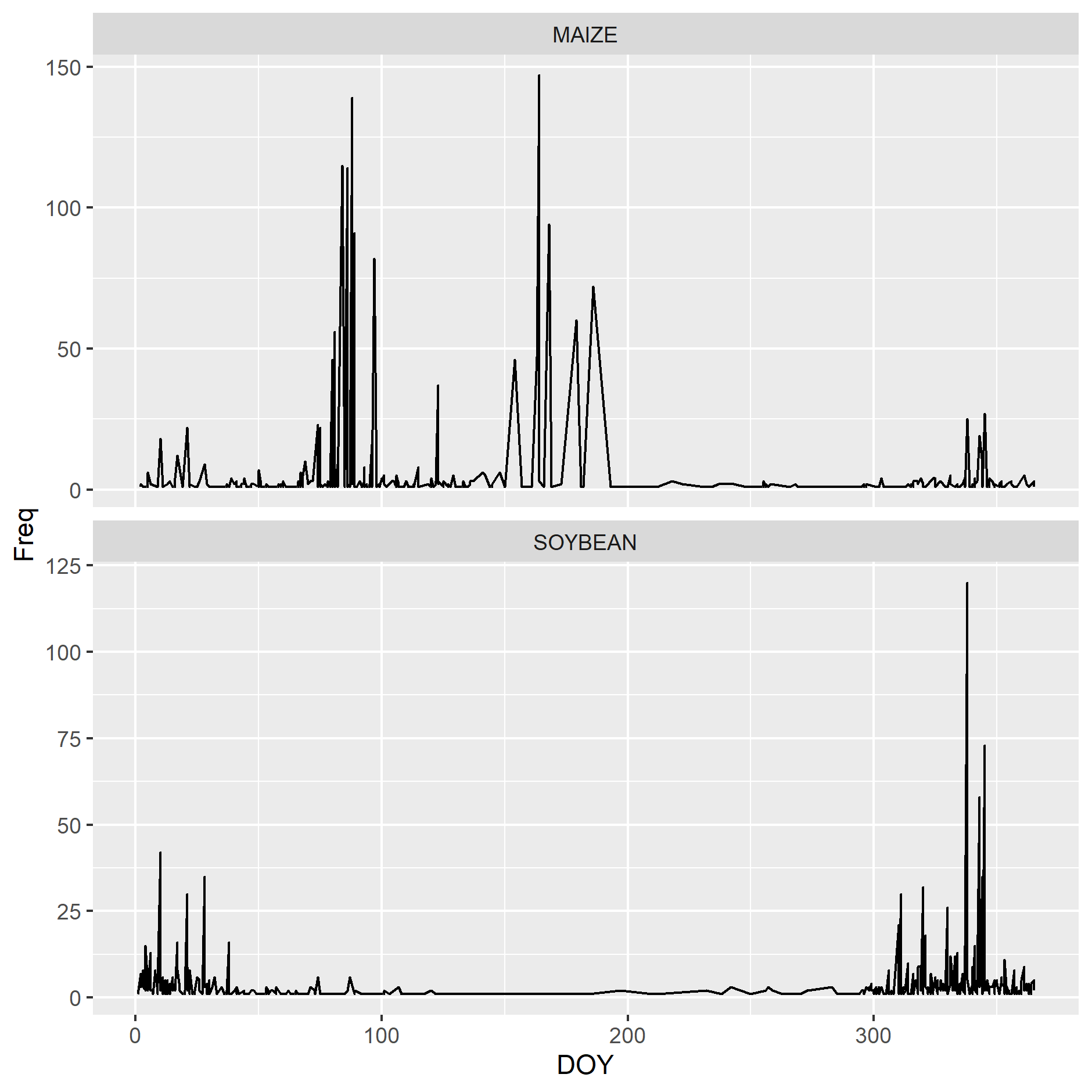
**

SP-Figure 3: Frequency of images by day of the year (DOY) predicted as showing diseased maize or soybean plants and taken by *Plantix* users or agronomists in Mato Grosso and Pará, between Nov 2016 and May 2020.
